# Treatment adherence and short-term outcomes of smoking cessation outpatient clinic patients

**DOI:** 10.18332/tid/94212

**Published:** 2018-08-30

**Authors:** Dilek Karadoğan, Özgür Önal, Deniz Say Şahin, Yalçın Kanbay, Sebih Alp, Ünal Şahin

**Affiliations:** 1 Department of Chest Diseases, Faculty of Medicine, Recep Tayyip Erdoğan University, Rize, Turkey; 2 Department of Public Health, Süleyman Demirel University, Isparta, Turkey; 3 Department of Social Services, Faculty of Economics and Administrative Sciences, Burdur Mehmet Akif Ersoy University, Burdur, Turkey; 4 Department of Psychiatric Nursing, School of Health Science, Çoruh University, Artvin, Turkey

**Keywords:** smoking, treatment adherence, quit success

## Abstract

**INTRODUCTION:**

Previous studies have shown that adherence to treatment is fundamental to success in smoking cessation. However, smoking cessation medication regimens are limited significantly by the struggle to adhere to them. This study was conducted to evaluate the factors associated with treatment adherence and quitting success in a group of patients that applied to our smoking cessation outpatient clinic (SCC).

**METHODS:**

Patients that applied to SCC between April 2015 and December 2016 who were evaluated, found suitable for smoking cessation interventions and started pharmacological treatment were included in this study. Only those who could be reached by phone three months after their first application became participants. Those who had used the prescribed treatment for at least 30 days were grouped as treatment-adherent.

**RESULTS:**

In total, data for 346 patients were evaluated. Mean (±SD) age was 44.3±13.9 years; most of them were male (63%), primary school graduated (36.1%), self-employed (43.7%), and had no comorbid diseases (71%). Bupropion was started in 52% of the patients, that rate was 35.8% for varenicline and 12.1% for a combination of the nicotine patch and gum. Mean days for treatment use was 20.9±18.5; 59% of the patients were non-adherent to their treatment and 51.7% had only one control visit number. Adverse reactions due to treatment were recorded in 25% of participants, and at their third month 37.9% of them had quit smoking. In multivariate logistic regression analysis, increase in control visit number, absence of adverse reaction, and varenicline use, were each associated with higher treatment adherence (p<0.001) and only being in the treatment-adherent group was associated with quit success (OR=3.01, 95% CI: 1.88–4.81, p=0.001).

**CONCLUSIONS:**

This study showed that most patients did not use their prescribed SC treatments adequately; a main factor that affects quit success is treatment adherence. There is a need for closer monitoring and follow-up to ensure adequate use of treatment of patients.

## INTRODUCTION

Smoking is a major cause of preventable early deaths worldwide^1^. Therefore, for a smoker, quitting smoking is fundamental to a chance for a longer and better life^2,3^. Most smokers, even at an early age, begin to perceive a worsening of their health and quality of life^4^. Most intend or attempt to quit at some point in their lives but are not successful^5^. It has been proven that quitting success is higher with assistance than without. Furthermore, counseling for behavioral therapy plus pharmacotherapy is more effective than pharmacotherapy alone^6^. In the last decade, smoking cessation outpatient clinics have become active, recommending combination therapy for smoking cessation according to guidelines in Turkey, the location of the current study. Turkey joined the World Health Organization (WHO) Framework Convention on Tobacco Control in 2005^7^. By signing and implementing all of the MPOWER policies, overall smoking prevalence in Turkey among individuals over 15 years of age decreased significantly: from 31.2% in 2008 to 27.1% in 2012^8^. Nationwide anti-tobacco policies included avoiding secondhand smoke by eliminating smoking in all indoor workplaces and public places. Also, the number of specified outpatient smoking cessation clinics increased in the last decade; until 2002 there were 25 in the whole country^9^. Now, in 2018, that number is over 400. Additionally, in 2011 the Turkish Ministry of Health (MoH) fist distributed 0.3 million varenicline and bupropion drugs to outpatient smoking cessation clinics (SCCs), countrywide and free of charge^10^. In 2011, the Turkish MoH conducted a program supporting individuals via free medications^11^. A study was conducted to evaluate the outcome of that free medication period in the first year, 2011; it was reported that willingness and adherence to treatment were key points for success in smoking cessation. While treatment non-adherence is a main factor for the gap between quit success and failure, previous studies have also identified non-adherence to medication as an important predictor of smoking relapse^12,13^ and also for abstinence^14^.

In randomized clinical trials, adherence to tobacco-dependence treatments were found^12^ to be over 98%, however that rate is around 25–30% in most real life settings^13,15^. There is one exception: in special comorbid groups, such as hospitalised patients for myocard infarction, that rate increases^16^ to 40%.

One of the factors influencing the use of smoking cessation medications is their cost, a particular barrier for patients with a low socioeconomic status^13^. In 2015 began a second period of free distribution of medications. It covered each smoker’s cessation medication for 3 months. Our current study evaluates the patients who applied in that second period. Our reasoning for limiting the study to that period was our belief that it is important to evaluate the treatment adherence of patients during the free period, to evaluate the effect that minimizing the cost barrier has. Our aim was to evaluate patients’ treatment adherence during the free medication period and evaluate their quit status at the end of the period. We also wanted to determine and measure the effectors for those outcomes. By evaluating quit status at the third month, instead of long-term or quit status at 12 months, we eliminate other potential factors that could affect quitting and relapses outside of the free treatment.

In this study we aimed to assess adherence to tobacco dependence treatment during a 3-month free varenicline and bupropion distribution period, and the factors associated with adherence. A second aim was to assess quit status/smoking abstinence status at the end of the treatment period, i.e. 3 months after beginning the first dose. Our findings will be an important reference for identifying gaps in national tobacco policies, measuring quantitatively the success of free medication periods, and developing new implementations for smoking cessation clinics.

## METHODS

### Setting and participants

The study population consisted of patients who had applied to a smoking cessation outpatient clinic located in the Eastern Black Sea region of Turkey. This SCC had accepted patients one day per week since July 2014 and was staffed with one pulmonologist who was certified and specialized in smoking cessation interventions. At the first visit, patients’ medical histories, physical examinations, pulmonary function tests (PFTs), chest x-rays, and laboratory blood analysis were evaluated, and patients were examined to determine whether or not they were appropriate candidates for smoking cessation interventions. The Fagerström test was also administered at this time, and not again throughout the rest of the study. Collected data regarding patients’ health status were recorded on a national electronic database called the ‘Tobacco Addiction Treatment Follow-up System’. In that electronic system, the first line of questioning included questions regarding patients’ demographic characteristics: age, gender, education level, and job title. The other sections covered: the Fagerström test of nicotine addiction level, which was administered to all patients at the baseline visit; comorbid diseases (through the questions ‘Do you have any other disorders? Do you use any medication?’); tobacco use history (‘How long have you been smoking?’); number of previous quit attempts and methods used (‘How many times you tried to quit, and with which method?’); and respiratory system complaints (‘Do you have cough, sputum, dyspnea, or wheezing?’). Blood pressure was also measured and recorded at this time. After recording this needed information, brief behavioral interventions based on clinical practice guidelines were also provided^17^ and afterwards appropriate treatment was offered to the patients, including the prescription of medication, if necessary, and setting a quit date. All data were recorded by interviewing the pulmonologist and patients.

In April 2015, the Turkish MOH began a second free medication period, based on the success of the period in 2011. During that time, smoking cessation medications (oral bupropion SR and varenicline) were distributed to SCCs by the Turkish MoH. Patients who started treatment within that period got the medications free of charge. They were informed about how to use the medication and advised of any potential adverse effects, which were also written in the patient-signed informed consent form. Then, the target quit date (TQD) was decided with patients, generally set to be 14 days after that same baseline visit. Patients were advised to use the medications regularly and to decrease the cigarettes smoked per day until the second visit, their TQD.

At the baseline visit, patients were given appointments for a second control visit 14 days later. At this second visit, patients were evaluated for any drug adverse reactions (‘Do you have any side effects due to the medication? What were the side effects?’) and asked about their daily smoking habits (‘How many cigarettes do you smoke in a day?’); abstinence status (‘When did you smoke your last cigarette?’); and readiness to quit (‘Are you ready to quit smoking today?’). Patients were again provided brief behavioral interventions and the next control visit date was planned, which was usually 2 weeks after this second visit. The same procedures used at the second control visit were administered at all subsequent visits. Those follow-up visits occurred at 2–3 week intervals until enough time had passed that all three monthly treatments of the free smoking medication could be used. Medication was covered for 3 months for each patient in a year.

Inclusion criterias were: 1) Adult smokers (older than 18 years old and ≥10 cigarettes/day over the last year and no period of smoking abstinence longer than 3 months in the past year), 2) being in good general health and having no history of bipolar disorder or schizophrenia and no current major depression and no alcohol abuse, 3) reached by phone calls at their third month from the baseline visit. Those who did not fall into this category or could not be reached by phone at the third month were excluded.

In total, 395 patients applied to our smoking cessation outpatient clinic between 1 April 2015 and 28 February 2016. Due to comorbid conditions-schizophrenia (5 patients), current major depression (1 patient), history of bipolar disorder (3 patients), or alcohol abuse (2 patients)-11 patients did not start medication and therefore were excluded from the study. After evaluations, 384 were deemed suitable for cessation treatment; they were approved to quit smoking and prescribed medications. Their name, telephone number and identity number were recorded from our registry notebook, and other demographic data and the control visit information were recorded from the electronic tobacco control system mentioned above. Three months after their first application they were called by phone. In total, 346 patients (90.1%) were reached; 38 patients could not be reached by phone. Based on self-report they were grouped as quitters or non-quitters. After the baseline visit, patients who had used the advised treatment for at least 30 days were grouped as treatment-adherent, and the rest were grouped as non-adherent.

Before starting the study, ethical approval from Artvin Çoruh Universiy Rectorship was obtained, also permission for the study was obtained from the Hospital’s General Secretary.

### Data collection

Sociodemographic characteristics of the patients (age, gender, education level, and occupation), comorbid diseases, Fagerström test score, treatment choice, and number of control visits, were obtained from information gathered at the baseline visit for each patient and then entered into the mandatory national electronic system. From the telephone interviews, information regarding the treatments’ effectiveness, any adverse reactions and their final smoking situation were evaluated. Final status was recorded for 2 categories: 1) quit smoking successfully after the planned quit day and still not smoking, and 2) could not quit after the planned quit day. Those who had quit on the planned quit day but relapsed were also classified under the non-quitter category.

### Statistical analysis

Analysis was done using Statistical Package for the Social Sciences, Version 20. We used simple descriptive statistics to characterize the study population and presented continuous data as mean (±SD) and categorical data numerically (%). To evaluate the effect of each characteristic (age, gender, education level, occupation, Fagerström test score, comorbid disease, start date of cessation medication, adverse reaction status, and number of control visits) on treatment adherence or quit status, univariate analysis was performed first. Afterwards, all of these variables were analyzed together for any possible association or interaction using multivariate regression analysis with a Backward LR test. The model was found to fit the data for treatment adherence: Backward Stepwise (likelihood ratio): -2 LOG likelihood: 406.928, Nagelkerke R Square: 0.220, Omnibus tests of model coefficient: p=0.000, Durbin-Watson test: 2.20. It also fit the data for quit status: Backward Stepwise (likelihood ratio): -2LOG likelihood: 416.276, Nagelkerke R Square: 0.158, Omnibus tests of model coefficients: p=0.000, Durbin-Watson test: 2.20. Results are presented as odds ratios and 95% CIs. A p-value < 0.05 was considered as significant.

## RESULTS

In total, data for 346 patients were evaluated. Mean (±SD) age of participants was 44.3±13.9 years, with males the majority (63.9%). Of the participants, 36.1% had graduated primary school. Detailed sociodemographic characteristics of the study population are shown in Table 1.

**Table 1:** Characteristics of the study population, Hopa State Hospital, 2015–2016 (n=395 )

**Age (Mean±SD)**	44.3±13.9 years (min 19, max 81)
	
**Gender**	
Female	125 (36.1%)
Male	221 (63.9%)
**Education level**	
Less than 5 years	5 (1.5%)
5 years primary schooling	125 (36.1%)
3 years secondary schooling	34 (9.8%)
3 years high schooling	114 (32.9%)
University graduated	68 (19.7%)
**Occupation**	
White collar worker	84 (24.3%)
Blue collar worker or farmer	98 (28.3%)
Self employed	52 (43.7%)
Retired	53 (15.3%)
Student	12 (3.5%)
Housewife or not working	86 (24.9%)
**Comorbid diseases**	
Chronic pulmonary or cardiovasculary diseases	68 (19.7%)
Other chronic diseases	32 (9.2%)
No comorbid disease	246 (71.1%)
**Smoking duration (mean±SD)**	22.2 ± 13.1 years
**Fagerström score**	6.6 ± 2.0
**Previous quit attempts**	1.6 ± 1.6
**Started medical treatment**	
cNRT	42 (12.1%)
Varenicline	124 (35.8%)
**Bupropion**	180 (52.0%)
**Number of days for smoking cessation medication use**	20.9±18.5 (min 0,max 90), 0 day for 30 (8.7%) patients
0 days	30 (8.7%)
≥8 weeks	32 (9.2%)
**Treatment adherent**	142 (41.0%)
**Treatment non-adherent**	204 (59.0%)
**Number of control visits (mean±SD)**	1.5 ± 1.1
0	31 (9.0%)
1	179 (51.7%)
2	85 (24.6%)
3	34 (9.8%)
4	9 (2.6%)
5	2 (0.6%)
6	6 (1.7%)
**Recorded medication adverse reactions**	
Present	88 (25.4%)
Absent	258 (74.6%)
**Final situation**	
Quit smoking	131 (37.9%)
Continuing smoking	215 (62.1%)
	346 (100%)

Most of the patients had no comorbid disease (71.1%). Mean smoking duration was 22.2±13.1 years, mean Fagerström test score was 6.6±2.0. Most patients were prescribed bupropion (52.0%). Mean days of medication use were 20.9±18.5. Most of them were non-adherent to their prescribed medication (59.0%). Most of them returned for only one follow-up visit (51.7%), most recorded no adverse reaction to prescribed medications (74.6%), and at their third month 37.9% had quit smoking (Table 1). Cessation was evaluated after a 3-month period, as opposed to the traditional at 12 months, for complete cessation, because the Turkish government no longer provides medications for cessation after that time.

Table 2 shows the relationship between each factor and treatment adherence. Bupropion and varenicline use were positively associated with treatment adherence compared to NRT, both in univariate and multivariate analysis. Also, varenicline use was 2.19 times more associated with treatment adherence compared to bupropion as a reference group in multivariate analysis. Absence of adverse reactions was positively associated with treatment adherence in both univariate and multivariate analysis, and higher visit numbers were positively associated with treatment adherence.

**Table 2:** Factors associated with treatment adherence in univariate and multivariate analyses, Hopa State Hospital, 2015–2016 (n=395 )

	*Univariate analysis*			*Multivariate analysis (Backward: LR)*				
	*OR*	*95% CI*	*p*	*OR*	*95% CI*	*p*
**Age group** (years)						
15–44	1					
45–64	1.365	0.647–2.881	0.414	–	–	NS
≥65	1.437	0.670–3.082	0.351			
**Gender** (woman compared to man)	1.251	0.798–1.961	0.328	–	–	NS
**Education level** (per 1 level increase)	1.070	0.891–1.285	0.470	–	–	
**Occupation** (working actively compared	0.951	0.618–1.465	0.821	–	–	NS
to not working)						
**Fagerström test score** (per 1 score	0.929	0.832–1.038	0.195	–	–	
increase)						
**Comorbid disease** (presence compared	1.187	0.741– 1.889	0.476	–	–	NS
to absence)						
**Started treatment choice**						
cNRT	1			1		
Bupropion	3.908	1.566–9.753	**0.003**	5.185	1.929–13.935	**0.001**
Varenicline	6.610	2.599–16.810	**0.001**	8.987	3.258–24.787	**0.001**
**Started treatment choice**						
Bupropion	1					
cNRT	0.25	0.103–0.639	**0.003**	Not adjusted in multivariate analysis				
Varenicline	1.69	1.065–2.685	**0.026**					
**Adverse reaction** (absence compared to presence)	2.091	1.238–3.532	**0.006**	3.326	1.868–5.924	**0.001**
**Control visit number** (per 1 visit increase)	1.599	1.283–1.993	**0.001**	1.708	1.353–2.156	**0.001**

Backward Stepwise (likelihood ratio): -2 LOG likelihood: 406.928. Nagelkerke R Square: 0.220. Omnibus tests of model coefficient: p=0.000. Durbin-Watson test: 1.96

Both middle aged and elderly groups had higher association with quitting success compared to younger groups in univariate analysis, while in multivariate analysis only the rates of middle-aged groups were statistically significantly higher than for young groups. Increased control visit numbers were positively associated with quitting success only in univariate analysis. Treatment adherence was positively associated with quitting success both in univariate and multivariate analyses (Table 3).

**Table 3:** Factors associated with successfull quit attempt in univariate and multivariate analyses, Hopa State Hospital, 2015–2016 (n=395)

	*Univariate analysis*			*Multivariate analysis (Backward: LR)*		
	*OR*	*95% CI*	*p*	*OR*	*95% CI*	*p*
**Age group** (years)						
15–44	1			1		
45–64	2.645	1.239–5.647	**0.012**	2.700	1.221–5.971	**0.014**
≥65	2.202	1.019–4.759	**0.045**	2.138	0.956–4.784	0.064
**Gender** (woman compared to man)	1.195	0.758–1.885	0.443	–	–	NS
**Education level** (per 1 level increase)	0.969	0.805–1.166	0.736	–	–	NS
**Occupation** (working actively compared to not working)	0.913	0.589–1.414	0.683	–	–	NS
**Fagerström test score** (per 1 score increase)	0.894	0.552– 1.448	0.649	–	–	NS
**Comorbid disease** (presence compared to absence)	1.065	0.953–1.190	0.264	–	–	NS
**Started treatment choice**						
cNRT	1					
Bupropion	1.143	0.571–2.288	0.706	–	–	NS
Varenicline	0.852	0.416–1.746	0.662			
**Adverse reaction** (absence compared to presence)	0.980	0.594–1.614	0.936	–	–	NS
**Control visit number** (per 1 visit increase)	1.305	1.067–1.596	**0.009**	–	–	NS
**Treatment adherent** (adherent compared to non-adherent)	1.599	1.283–1.993	**0.001**	3.231	2.036–5.129	**0.001**

Backward Stepwise (likelihood ratio): -2LOG likelihood: 416.276. Nagelkerke R. Square: 0.158. Omnibus tests of model coefficients: p=0.000. Durbin-Watson test: 2.20

Mean days of prescribed medication use was lower in the non-quitter group than quitter group (16.2±16.3 vs 28.5±19.3, p=0.001). The most common adverse reactions were nausea (30 patients), dizziness (12 patients) and dry mouth (11 patients).

## DISCUSSION

This study shows that most of the patients were non-adherent to their prescribed medications, did not follow their control visits appropriately, and at the third month had a quit rate of 37.9%. Varenicline and bupropion use had positive associations with treatment adherence, while cNRT use had negative association. Additionally, varenicline use had significantly higher association with treatment adherence than either bupropion or cNRT. Absence of adverse reactions and increased office visits were also positively associated with treatment adherence. Older age, increased office visits, and treatment adherence were in turn positively associated with quit success.

These findings indicate that for successful outcomes of smoking cessation interventions, patients’ adherence to cessation programs plays the key role. Therefore, addressing and discussing the factors affecting adherence is of fundamental importance to improving smoking cessation programs. While there is no standardized description of treatment adherence for SC interventions in the literature^18^, in previous studies it has usually been classified according to duration of medication use or follow-up group therapies^19,20^, as we did in our study. There is a need for a standardized description that includes duration of medication use as well as status of follow-up visits. While guidelines advise for at least 8 weeks of treatment^17,21^, in our study we used a 30-day cut-off period to determine treatment adherence; the distributed medications were packaged monthly and we asked each participant to finish an entire pack. Our results showed that our patients’ mean medication use was 20.9±18.5 days; 59% used it for under 30 days (classified as non-adherent to treatment), 8.7% never used the advised treatment, and only 32 patients (9.2%) used the medication for 8 weeks and over. A single-center study that evaluated the effectiveness of smoking cessation interventions in 2011, during the first free medication period in Turkey, found that 3.95% of the patients that were prescribed SC medication never used it^20^. The view was that the smokers were neither willing nor ready to quit. This is also a possible explanation for the current study, which also took place during a free medication period. Some of the patients most likely just rushed to get free medication, because the period is generally only a year or a year and a half. Clinician evaluations for intentions and stage of motivation for quitting would be helpful so that patient education is more effective. Another suggestion is a government policy that would require covering SC medications under health insurance, thus allowing smokers to be able to apply whenever they feel ready to quit.

In 2011, during the first period of free medication (varenicline and bupropion SR) distribution in Turkey, a nationwide study was conducted to evaluate participant outcomes after twelve months in the Nationwide Smoking Cessation Treatment Support Program^11^. In that study, researchers evaluated the factors affecting quitting and abstinence success. Overall quit rate was 27.9% in their study, with higher quit rates among varenicline users. In our study, we evaluated patients at their third month and found the quit rate to be 37.9%. In other national studies, the quit rate in the short-term was found to be 25% on average^20,22^. However, in international studies that rate was found to be higher^23,24^ or similar^25^. In our SCC, the same pulmonologist always gave the counselling and follow-ups; additionally, there were patients that applied after the implementation of 5A and 5R procedures^26^ by the same doctor. Such factors could possibly contribute to this study having a higher quit rate than other national studies. However, the limited number of close follow-ups and phone contacts, as well as limited extended pretreatment and patient education, may explain the lower rates than in international studies^27^. If that were the case, more effort and staff are needed for a better outcome in Turkey.

Varenicline users’ quit rate was not statistically significantly different from the other treatment choices, both in univariate and multivariate analyses in our study. However, varenicline’s effect on treatment adherence was higher than that of bupropion SR and cNRT in both analyses. Also, the bupropion SR group’s treatment adherence was higher than for cNRT. In a review^28^, combination NRT adherence was found to be higher compared to other drugs. However, when offered over-the-counter, NRT was found to have lower efficacy compared to varenicline and bupropion due to premature discontinuation^13^. Most of the reasons for premature discontinuation fall into the following categories: relapsing back to smoking, experiencing side effects, believing that the medication was no longer needed, and wanting to avoid the cost^13^. In our clinical experience, our patients have also mentioned those types of reaction; however in this study we did not record such complaints. In our study, cNRT’s low adherence rate can also be explained by the coverage status—nicotine patches and gums might be difficult for smokers to afford. These barriers decrease treatment adherence, therefore the government’s policy of free application should include all forms of FDA-approved SC medications. Other reasons for early discontinuation should also be evaluated in order to find solutions.

Among patient characteristics, age was found to be one of the factors for quit success in our study. Older ages were associated with higher quit success, even if they did not use adequate amounts of the medications. This result is in accordance with previous studies that found older adults to have a higher quit rate^5,29^.

In other national studies, Benli et al.^20^, for example, found that ‘side effects’ were the most common reason for early discontinuation. In our study, factors that affected treatment adherence were the presence of adverse reactions and the number of follow-up visits. Absence of adverse reactions was positively associated with treatment adherence. Mostly minor side effects were reported; among them nausea had the highest rate. Overall rate of patients with adverse reactions was 25.9%. We believe that overcoming adverse reactions would be possible with regular visits and patient education. Therefore, more close follow-ups, even by phone, are required. There is a free national quit-line called ‘ALO 171’ in Turkey. Smokers are able to reach out when they need assistance, through that quit-line they can also arrange their SCC appointments and get counselling. Patients using the quit-line are called by phone, first at the planned quit date; then after 1 week, 1 month, 3 months, 6 months, and 12 months, all counted from the planned quit date. That call is intended to provide motivational support, however our opinion is that each SCC should also have a team consisting of staff, familiar to the patients, who can give individualized phone support. That level of support would be more effective. Before starting treatment, patients should also be educated, not only about the side effects of the drugs but also about the mechanisms and how they will work during the cessation process. The benefit of that kind of intervention has been studied previously^29^. Therefore, there is a need for both a more professional follow-up team and longer periods of cessation efforts.

Lower treatment use is the main problem, and factors that affect this result should be evaluated and studied to find solutions that will be clinically relevant. Also, great care should be taken to choose only patients with a sincere intention to quit, and only they should be treated for SC. The others, who are not ready and are only applying to get the drug for use at another time should be educated and persuaded to apply when they are ready. On this issue, government policy on tobacco addiction plays an important role. Because medications were again distributed for free for only short periods of time, smokers rushed to get the medication, even though they did not plan to quit but just to have them for future use. At admission, 60 tablets of medication were given to patients—enough for 1 month. That is one of the reasons our study chose 30 days as the cut-off period for treatment adherence. As seen in our results, 30 patients had not even started using medication by that time. Mean medication use is under 30 days and more than half of the patients came to follow-up visits only once. Our suggestion is that weekly packaging and distribution of drugs will be an effective way to increase follow-up visits. Patients may be more willing to attend control visits in order to receive their medications. Instead of monthly packs, weekly packs allow the doctor to prescribe an adequate amount of medicine until the planned control visit date. Also, governments that offer free medication coverage should include all forms of FDA-approved SC medications, not just periodically but also regularly. Additionally, Turkey has a need for comparison studies between both period outcomes.

The study design—the real-life clinical experience of patients who underwent smoking cessation interventions by the same clinician—makes the study strong. However, we did not use any biological confirmation of smoking status, instead grouping patients according to their report. This is one of the limitations of the study. Also, we did not record the patient-related reasons for non-adherence with the medications. Cessation in this study was measured after three months; it would be valuable for future studies to follow up one year after the participant’s quit date to evaluate whether or not cessation was ultimately successful.

For a higher quit rate, intention to quit should be measured objectively in order to start the SC intervention at the best time for the patient. For the effect of coverage status, SCMs should also be evaluated; there is no comparison study on that topic in Turkey. There is also a need for qualitative studies to evaluate the reasons for not using medications adequately. Similarly, long-term follow-up experiences are needed to gain more data on quit success.

## CONCLUSIONS

The current study evaluated treatment adherence and quit success of patients admitted to a Turkish SCC. The results showed that treatment adherence is the main factor in quit success. However, most of the patients were non-adherent to their prescribed SC medications. Additional research and interventions such as covering all kinds of approved SCMs, changes in packaging and distribution, and closer, more individualized follow-ups for motivation and support in overcoming minor drug-related side effects are required; all of these implementations would increase treatment adherence and in turn result in a higher quit success.

## References

[1] World Health Organization Tobacco 2015.

[2] Baker CL, Flores NM, Zou KH, Bruno M, Harrison VJ (2017). Benefits of quitting smoking on work productivity and activity impairment in the United States, the European Union and China. Int J Clin Pract.

[3] (2017). Quitting smoking: the benefits. Breathe.

[4] Çakmakçı Karadoğan D, Önal Ö, Say Şahin D, Yazıcı S, Kanbay Y (2017). Evaluation of school teachers’ sociodemographic characteristics and quality of life according to their cigarette smoking status: a cross-sectional study from the eastern Black Sea region of Turkey. Tuberk Toraks.

[5] El-Khoury Lesueur F, Bolze C, Melchior M (2018). Factors associated with successful vs. unsuccessful smoking cessation: Data from a nationally representative study. Addict Behav.

[6] Zhu S, Melcer T, Sun J, Rosbrook B, Pierce JP (2000). Smoking cessation with and without assistance: a population-based analysis. Am J Prev Med.

[7] World Health Organization Tobacco Control Country Profiles 2014.

[8] Bilir N, Özcebe H (2013). Tobacco control activities in Turkey. Turk J Public Health.

[9] Kunt Uzaslan E, Örsel O, Demir T Türkiye’de Sigara Bıraktırma Poliklinikleri.

[10] Elbek O, Kılınç O, Aytemur ZA (2015). Tobacco Control in Turkey. Turk Thorac J.

[11] Çelik İ, Yüce D, Hayran M (2015). Nationwide Smoking Cessation Treatment Support Program--Turkey project. Health Policy.

[12] Hays JT, Leischow SJ, Lawrence D, Lee TC (2010). Adherence to treatment for tobacco dependence: association with smoking abstinence and predictors of adherence. Nicotine Tob Res.

[13] Balmford J, Borland R, Hammond D, Cummings KM (2011). Adherence to and reasons for premature discontinuation from stop-smoking medications: datafrom the ITC Four-Country Survey. Nicotine Tob Res.

[14] Schlam TR, Cook JW, Baker TB (2018). Can we increase smokers’ adherence to nicotine replacement therapy and does this help them quit?. Psychopharmacology.

[15] Liberman JN, Lichtenfeld MJ, Galaznik A (2013). Adherence to varenicline and associated smoking cessation in a community-based patient setting. J Manag Care Pharm.

[16] Grandi SM, Eisenberg MJ, Joseph L, O’Loughlin J, Paradis G, Filion KB (2016). Cessation treatment adherence and smoking abstinence in patients after acute myocardial infarction. Am Heart J.

[17] 2008 PHS Guideline Update Panel, Liaisons, and Staff (2008). Treating tobacco use and dependence: 2008 update U.S. Public Health Service Clinical Practice Guideline executive summary. Respir Care.

[18] Pacek LR, McClernon FJ, Bosworth HB (2017). Adherence to pharmacological smoking cessation interventions: A literature review and synthesis of correlates and barriers. Nicotine Tob Res.

[19] Figueiró LR, Barros HM, Ferigolo M, Dantas DC (2017). Assessment of factors related to smokers’ adherence to a short-term support group for smoking cessation: a longitudinal study in a developing country. Trends Psychiatry Psychother.

[20] Benli AR, Erturhan S, Oruc MA, Kalpakci P, Sunay D, Demirel Y (2017). A comparison of the efficacy of varenicline and bupropion and an evaluation of the effect of the medications in the context of the smoking cessation programme. Tob Induc Dis.

[21] Patnode CD, Henderson JT, Thompson JH, Senger CA, Fortmann SP, Whitlock EP (2015). Behavioral Counseling and Pharmacotherapy Interventions for Tobacco Cessationin Adults, Including Pregnant Women: A Review of Reviews for the U.S. Preventive Services Task Force [Internet]. Report No.: 14-05200-EF-1. U.S. Preventive Services Task Force Evidence Syntheses, formerly Systematic Evidence Reviews.

[22] Önür ST, Uysal MA, İliaz S (2016). Does Short Message Service Increase Adherence to Smoking Cessation Clinic Appointments and Quitting Smoking?. Balkan Med J.

[23] Trofor AC, Man MA, Marginean C, Dumitru F, Trofor L (2016). Smoking cessation for free: outcomes of a study of three Romanian clinics. Open Med.

[24] Gonzales D, Rennard SI, Nides M (2006). Varenicline, an alpha4beta2 nicotinic acetylcholine receptor partial agonist, vs sustained-release bupropion and placebo for smoking cessation: a randomized controlled trial. JAMA.

[25] Rosen LJ, Galili T, Kott J, Goodman M, Freedman LS (2018). Diminishing benefit of smoking cessation medications during the first year: a meta-analysis of randomized controlled trials. Addiction.

[26] World Health Organization (2014). Toolkit for delivering the 5A’s and 5R’s brief tobacco interventions in primary care.

[27] Hollands GJ, McDermott MS, Lindson-Hawley N, Vogt F, Farley A, Aveyard P (2015). Interventions to increase adherence to medications for tobacco dependence. Cochrane Database Syst Rev.

[28] Baker TB, Piper ME, Stein JH (2016). Effects of Nicotine Patch vs Varenicline vs Combination Nicotine Replacement Therapy on Smoking Cessation at 26 Weeks: A Randomized Clinical Trial. JAMA.

[29] Kim Y, Cho WK (2014). Factors Associated with Successful Smoking Cessation in Korean Adult Males: Findings from a National Survey. Iran J Public Health.

